# High prevalence of NTRK fusions in sporadic dMMR/MSI mCRC RAS/RAF wild-type: an opportunity for a post-immune checkpoint inhibitors progression rescue strategy

**DOI:** 10.1016/j.esmogo.2024.100084

**Published:** 2024-07-16

**Authors:** M. Svrcek, A. Cayre, T. Samaille, R. Colle, L. Mas, P. Bourgoin, E. Guillerm, R. Cohen, F. Penault-Llorca, T. André, N. Radosevic-Robin

**Affiliations:** 1 Department of Pathology, Sorbonne University, Saint-Antoine Hospital, AP-HP, Paris; 2 Department of Genetics, Functional Unit of Onco-Angiogenetics and Genomics of Solid Tumors, AP-HP, Sorbonne University, Pitié Salpêtrière Hospital, Paris; 3 Department of Medical Oncology, Sorbonne University, Saint-Antoine Hospital, AP-HP, Paris; 4 Sorbonne University, INSERM UMRS_938, Microsatellite Instability and Cancer, Saint-Antoine Research Center, Paris; 5 Department of Pathology, Clermont Auvergne University, INSERM U1240, Centre Jean Perrin, Clermont-Ferrand, France

**Keywords:** tropomyosin receptor kinase, mismatch repair deficient, metastatic colorectal cancer, immune checkpoint inhibitors

## Abstract

**Background:**

Currently, mismatch repair deficient/microsatellite instable (dMMR/MSI) status constitutes a validated predictive marker of response to immune checkpoint inhibitors (ICIs) in patients with metastatic colorectal cancer (mCRC). Unfortunately, some of these patients do not benefit from ICIs. Very limited therapeutic options for patients with dMMR/MSI mCRC after progression on ICI(s) exist. These patients show poor outcomes with conventional chemotherapy. Inhibitors of tropomyosin receptor kinase (TRK) have shown promising activity against neurotrophic tropomyosin receptor kinase (*NTRK*) fusion-driven cancers. *NTRK* gene fusions are rare (<1%) in CRC and data regarding their prevalence in patients with dMMR/MSI CRC are increased but limited, especially in patients with metastatic disease.

**Materials and methods:**

A total of 187 dMMR/MSI mCRC patients, including 120 immune ICI-treated, were screened for *NTRK* gene fusions.

**Results:**

Tumor samples of 10 (5.3%) patients harbored *NTRK* gene fusions confirmed by FISH (*NTRK1* = 8; *NTRK3* = 2) including two cases with Lynch syndrome and height sporadic cases with *MLH1* promoter hypermethylation and *RAS* wild-type (wt), out of which only five were positive by pan-TRK immunohistochemistry. One patient with primary resistance to nivolumab received a TRK inhibitor (larotrectinib) and showed a complete response.

**Conclusions:**

These results underline the importance of screening for *NTRK* gene fusions in dMMR/MSI mCRC in sporadic cases with *MLH1* promoter hypermethylation RAS/BRAF^V600E^ wt. We highlight several key technical aspects of *NTRK* fusion testing and interpretation of reports that remain to be explored.

## Highlights

• In this population, the global *NTRK* fusion incidence in MSI mCRCs was 5.3%.
• There were more *NTRK* fusion-carrying cases in MLH1/RAS/RAF wt subpopulation.
• MSI mCRCs with MLH1 hypermethylation/RAS/RAF wt should be routinely screened for *NTRK* fusions.
• TRK inhibitors provide targeted therapy for MSI tumors with NTRK fusion after ICI progression.

## Introduction

The incorporation of molecular profiling into therapy selection and the identification of actionable targets for patients with metastatic colorectal cancer (mCRC) have led to significantly longer survival. Currently, mismatch repair deficient/microsatellite instable (dMMR/MSI) status constitutes a validated predictive marker of response to immune checkpoint inhibitors (ICIs) in mCRC patients. Indeed, ICIs have demonstrated an impressive clinical activity amongst dMMR/MSI mCRC patients in several non-randomized phase II trials in advanced lines and in the randomized first-line study, with durable clinical responses in heavily pretreated patients.^1^, ^2^, ^3^, ^4^, ^5^ Some dMMR/MSI mCRC patients do not benefit from ICIs and those who progress on ICI have very limited treatment options and poor outcomes on conventional chemotherapies.^1^, ^2^, ^3^, ^4^, ^5^, ^6^

Recently, inhibitors of tropomyosin receptor kinase (TRKi) have shown promising activity against neurotrophic TRK (*NTRK*) fusion-driven cancers.^7^^,^^8^ Consequently, larotrectinib and entrectinib became the first and the second tumor-agnostic cancer treatment to be approved by the European Medicines Agency (2019 and 2020, respectively) in patients not amenable to surgery, with chemorefractory mCRC, and whose tumors display an *NTRK* gene fusion.^9^ *NTRK* genes, which consist of *NTRK1-3* encoding TRK-A, TRK-B, and TRK-C proteins, are a family of transmembrane tyrosine kinases that play a role in cell proliferation and resistance to anoikis and nervous system physiology. Aberrant activation of *NTRK* genes, predominantly by gene fusions involving kinase domain, acts as a driver of oncogenesis in various solid and hematopoietic tumors, including CRC. These oncogenic fusions occur when the 3' region of the kinase domain of *NTRK1*, *NTRK2*, or *NTRK3* gene fuses with any of a number of N-terminal partners, forming a chimeric gene/oncogene that expresses constitutively activated tyrosine kinase.^10^ The frequency of *NTRK* gene fusions is heterogeneous according to the tumoral type. Oncogenic *NTRK* fusions are rare, occurring in <1% of unselected CRCs. However, they are observed more frequently in dMMR/MSI CRCs with *MLH1* promoter hypermethylation (MLH1ph) and wild-type (wt) *RAS* and *BRAF*^*V*600^, with rates ranging from 17% to 44%.^11^, ^12^, ^13^, ^14^, ^15^, ^16^, ^17^, ^18^, ^19^, ^20^, ^21^, ^22^, ^23^ Interestingly, a marked and durable response to larotrectinib has been reported in patients with dMMR/MSI mCRC harboring an *NTRK* fusion after progression on ICIs, suggesting the potential for TRK inhibitors in this patient population for whom the need for innovative therapeutics is greatest, notably after failure of ICIs.^24^ However, data on the prevalence and clinicopathological characteristics of *NTRK* fusion-harboring tumors within this specific subgroup of dMMR/MSI mCRCs are limited.^13^^,^^14^ Moreover, the assessment of diagnostic tools and strategies to identify dMMR/MSI CRCs that could benefit from TRK inhibitor therapies have been mainly evaluated in non-metastatic patients.^19^, ^20^, ^21^

The aim of this study was to evaluate the rate of *NTRK* gene fusions in patients with dMMR/MSI mCRC, regardless of prior treatment with ICI(s). We evaluated several diagnostic tools for detecting NTRK fusions in this specific subgroup of dMMR/MSI mCRC, including immunohistochemistry (IHC), FISH, and targeted RNAseq.

## Materials and methods

### Ethics statement

This study was carried out in accordance with the Declaration of Helsinki and was approved by the ethics committee (N°2020 – CER 2020-6). Non-opposition was obtained from each patient included in the study.

### Patients and sample collection

Tumor samples (primary tumor and metastasis-matched or primary tumor alone and metastasis alone) of dMMR/MSI mCRC patients were obtained from a French multicenter retrospective cohort^25^ and a large single-center prospective cohort of consecutive patients treated with anti- programmed cell death protein 1 (PD-1) monotherapy or the anti-PD-1 plus anti-cytotoxic T-lymphocyte-associated protein 4 combination from February 2015 to June 2022 (‘Immuno-MSI French cohort’) at Saint-Antoine Hospital (Paris, France). Clinicopathological data, including *RAS* (including *KRAS* and *NRAS* mutation) and *BRAF*^*V600E*^ status, the lost MMR protein, and *MLH1* methylation status, were available for all patients. Patients were categorized as having Lynch syndrome (LS)-associated CRC only if a germline mutation was confirmed and were classified as having sporadic CRC only if they exhibited loss of MLH1/PMS2 protein expression associated with *BRAF*^*V600E*^ mutation and/or hypermethylation of *MLH1* promoter, or if presented with biallelic somatic mutations of MMR genes.

### IHC, FISH, and RNAseq

All samples were screened for TRK protein expression on tissue microarrays (three 0.6-mm-diameter cylinders per tumor) by IHC using a pan-TRK antibody [clone EPR17341 (Abcam, Cambridge, United Kingdom)] with positive run controls (neurons or/and the ganglia cells in a whole tissue section of the appendix, showing granular cytoplasmic staining). In addition, the same cells were sought for in each whole tumor tissue section assessed. Tumor positivity was defined as >1% of tumor cells displaying cytoplasmic, membranous, nuclear, and/or perinuclear staining. Samples were screened for *NTRK1*/2/*3* gene rearrangements by FISH [ZytoLight SPEC NTRK dual-color Break Apart probe (ZytoVision, Bremerhaven, Germany)]. The threshold of 10%-15% tumor nuclei showing the break-apart signal was considered indicative of gene rearrangement positivity.^24^ IHC- or FISH-positive cases on tissue microarrays were additionally assessed on whole tumor tissue sections. Targeted RNAseq [FusionPlex® (Invitae, San Francisco, CA) Lung SK0133 ARCHER kit] was carried out on cases positive for both IHC and FISH, as well as on doubtful cases showing faint homogeneous immunostaining, a clear break-apart signal with a percentage of positive cells below the threshold, and heterogeneous patterns on FISH.

### Role of the funding source

The funders were not involved in the design of the study; the collection, analysis, and interpretation of the data; the writing of the article; or the decision to submit the article for publication.

## Results

### Patient characteristics and NTRK fusion genes identification

**Figure 1:**
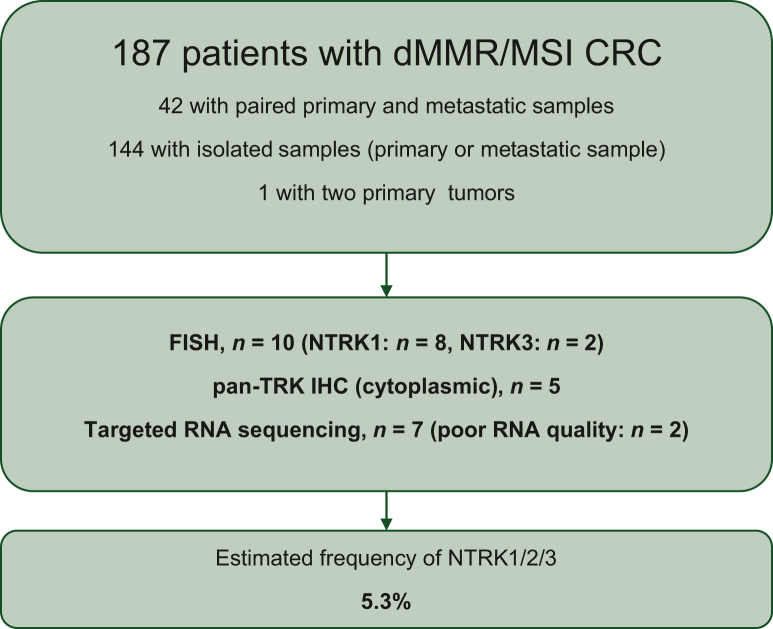
Flow chart. CRC, colorectal cancer; dMMR, mismatch repair deficient; IHC, immunohistochemistry; MSI, microsatellite instable.

**Figure 2:**
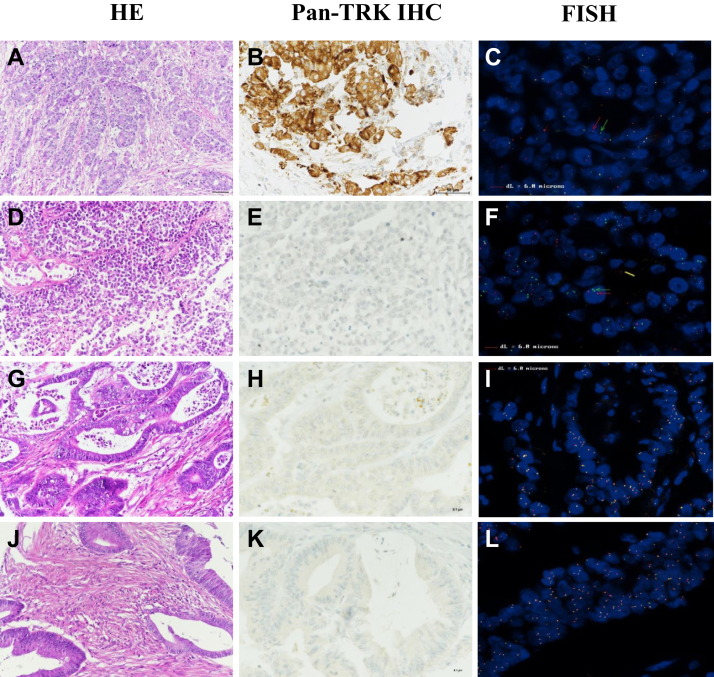
**High-resolution hematoxyline-eosine and immunohistochemistry and fluorescence in situ hybridization photos of NTRK cases.** (A) HE (×200 magnification, full section; scale bar: 50 μm). (B) Pan-TRK-positive IHC (×400 magnification, full section; scale bar: 50 μm) in one case displaying strong, diffuse cytoplasmic immunostaining. (C) This case harbored *NTRK1* fusion by FISH (×600 magnification), with 87.74% of positive nuclei with break-apart signals. (D) HE (×200 magnification, TMA spot). (E) Discordant case pan-TRK negative (×400 magnification, TMA spot) harboring. (F) *NTRK3* fusion (×600 magnification), with 99.00% of positive nuclei (splits red/green). (G) HE (×200 magnification, TMA spot). (H) One LS-related case pan-TRK negative (×400 magnification, TMA spot), exhibiting heterogeneous *NTRK1* rearrangement (I), with 15.88% of positive nuclei (splits red/green) (×600 magnification). (J) HE (×200 magnification, TMA spot). (K) The second LS-related case pan-TRK negative (×400 magnification, TMA spot), exhibiting heterogeneous *NTRK1* rearrangement (L), with 12.00% of positive nuclei (3' red isolated signals). HE, hematoxyline-eosine; IHC, immunohistochemistry; LS, Lynch syndrome; TMA, tissue microarray; TRK, tropomyosin receptor kinase.

A total of 187 patients (matched primary metastatic tumor samples, *n* = 42; one sample, *n* = 144; two primary tumors, *n* = 1) were screened (Figure 1 and Table 1). Of these, 120 received ICI-based treatment. Tumor samples from 10 (5.3%) patients harbored *NTRK* fusion genes as assessed by FISH (*NTRK1* = 8; *NTRK3* = 2), with five being pan-TRK IHC-positive. Sixteen cases with MLH1ph were *RAS*/*RAF* wt and 55 cases were LS with germinal mutation (Table 2). All LS cases showed moderate- to high-intensity cytoplasmic staining (Figure 2A-L). The Archer assay detected *TPM3-NTRK1* (*n* = 3), *LMNA-NTRK1* (*n* = 2), *ETV6-NTRK3* (*n* = 1), and *EML4-NTRK3* (*n* = 1) fusion transcripts in 7 out of the 10 patients, including all pan-TRK-positive cases (Figure 2). Eight cases were sporadic with *MLH1*ph (including seven *RAS/RAF* wt cases), and two *NTRK1*-rearranged cases had germline MMR mutations, corresponding to LS (Table 1), with loss of MSH2/MSH6 and isolated loss of MSH6 expression, respectively. Interestingly, heterogeneous *NTRK1* fusion FISH signals with a percentage of nuclei displaying undisputable break-apart signals close to the detection threshold (12.0% and 15.9%) and with <30% (25.9%) were detected in two patients with LS and in one sporadic case, respectively (Figure 2E-H). *NTRK1* rearrangements were not detected by the Archer assay in these cases due to poor RNA quality. One LS-related tumor was *KRAS-*mutated, and one sporadic tumor was *BRAF*^*V600E-*^mutated. The molecular characteristics of tumors harboring *NTRK* fusions are summarized in Table 1.

**Table 1:** Patient characteristics

Characteristics	All population (*N* = 187)	Patients with NTRK fusion (*n* = 10)
*RAS/BRAF*^*V600*^ molecular status		
*RAS/BRAF*^*V600*^ wild-type	64	8
*RAS* mutated	64	1
*BRAF*^*V600*^ mutated	45	1
Undetermined	13	0
Immunochemistry loss		
MLH1/PMS2	113	8
PMS2 alone	6	0
MSH2/MSH6	39	1
MSH6 alone	15	1
Undetermined	14	0
Hypermethylation MLH1 promoter (MLH1/PMS2 loss)		
Yes	48	8
No	38	0
Undetermined	27	0
Hypermethylated MLH1 + *RAS/BRAF*^*V600E*^ wild-type	16	7
Hypermethylated MLH1 + *RAS* wild-type and *BRAF*^*V600*^ mutated	1	1
Lynch status		
Sporadic	68	8
• Hypermethylated MLH1 or *BRAF*^*V600E*^Mutated	65	8
• Bi-allelic mutation	3	0
Germline mutation	55	2
Undetermined	64	0

NTRK, neurotrophic tropomyosin receptor kinase.

**Table 2:** Clinical and molecular characteristics of NTRK patients

Age, years	Sex	RAS	BRAF	MMR IHC	MLH1ph	Lynch status	Location	No. of met sites	Met sites	No. of treatment lines	ICI	Response to ICI	Survival, months	Pan-TRK IHC+	NTRK FISH	RNAseq: partner gene
**50**	F	Wt	Wt	MLH1/PMS2	Yes	Sporadic	Right	3	Lung, liver, peritoneum	2	0	NA	19	Yes	NTRK1	TPM3-TRK1-
**85**	F	Wt	Mut	MLH1/PMS2	Yes	Sporadic	Right + rectum	1	Peritoneum	1	0	NA	14	No	NTRK1	Neg^a^
**59**	F	Wt	Wt	MLH1/PMS2	Yes	Sporadic	Right	2	Peritoneum, lymph node	Uk	0	NA	Uk	No	NTRK1	TPR-NTRK1-
**58**	M	Wt	Wt	MSH2/MSH6	NA	Lynch	Right x2 + rectum	1	Liver	1 + local treatment	0	NA	96^b^	No	NTRK1	Neg^a^
**68**	M	Wt	Wt	MLH1/PMS2	Yes	Sporadic	Right	1	Lymph node	2	0	NA	14	No	NTRK3	ETV6-NTRK3
**75**	F	Wt	Wt	MLH1/PMS2	Yes	Sporadic	Left	1	Peritoneum	1 + local treatment	0	NA	78^b^	Yes	NTRK1	LMNA-TRK1
**76**	F	Wt	Wt	MLH1/PMS2	Yes	Sporadic	Left	3	Peritoneum, lymph node, liver	3	Anti-PD-1 + Anti-CTLA-4	CR	52^b^	Yes	NTRK1	LMNA-TRK1
**53**	F	Wt	Wt	MLH1/PMS2	Yes	Sporadic	Right	1	Lymph node	3	Anti-PD-1	PR	51^b^	Yes	NTRK3	EML4-NTRK3
**71**	F	Wt	Wt	MLH1/PMS2	Yes	Sporadic	Right	1	Peritoneum	3	Anti-PD-1	SD then PD	75^b^	Yes	NTRK 1	TMP3-TRK1-
**55**	M	Mut	Wt	MSH6	NA	Lynch	Right + left	3	Peritoneum, lymph node, liver	3	Anti-PD-1 + Anti-CTLA-4	CR	86^b^	No	NTRK1	Neg^a^

anti-CTLA-4, cytotoxic T-lymphocyte-associated protein 4; CR, complete response; CTLA-4, cytotoxic T-lymphocyte-associated protein 4; ICI, immune checkpoint inhibitor; IHC, immunohistochemistry; left, left-sided colon; met, metastatic; MLH1ph, MLH1 promoter hypermethylation; MMR, mismatch repair; Mut, mutated; NA, not applicable; Neg, negative; NTRK, neurotrophic tropomyosin receptor kinase; PD, disease progression; PD-1, programmed cell death protein 1; PR, partial response; right, right-sided colon; SD, stable disease; TRK, tropomyosin receptor kinase; uk, unknown; Wt, wild-type.

a Low-quality sample.

b Patients still alive.

### The association between NTRK fusion and clinical response in ICI-treated patients

Among the four *NTRK* fusion-positive patients who received ICI(s), three had objective responses by RECIST without disease progression (PD) at the time of writing and one developed primary resistance. The latter patient received an anti-PD-1 ICI (nivolumab) until confirmed peritoneal PD. This patient was included in the phase II NAVIGATE trial (NCT02576431) testing the efficacy of a TRK inhibitor (larotrectinib) in different types of cancers harboring *NTRK* fusions. larotrectinib administration at 100 mg twice daily started in May 2021, and as of March 2024, the patient remains on treatment, exhibiting good tolerance except intermittent diarrhea grade 1, an improvement in general condition (plays tennis) and weight (+5 kg), performance status 0, and maintenance of stable complete response during surgical laparotomy with restoration of digestive continuity.

Following this cohort, our center applied an algorithm to screen patients with dMMR/MSI CRCs with MLH1ph and *RAS* wt, and experiencing PD on ICI(s), using next-generation sequencing. Between January 2022 and March 2024, three patients with these tumoral characteristics were tested. One patient was identified with NTRK fusion (*TMP3-NTRK1* gene fusion) via RNAseq [*Idylla*™ *GeneFusion Assay* [Biocartis, Mechelen, Belgium]). This patient received anti-PD-1 therapy (pembrolizumab) until confirmed peritoneal PD and, subsequently, was included in the phase II NAVIGATE trial and received larotrectinib at 100 mg twice daily started in June 2022. A complete response was observed, and as of March 2024, the patient continues to receive the treatment.

## Discussion

Currently, routine *NTRK* fusion testing in CRC is not common practice given their rarity. Notably, considering that the frequency of *NTRK1/2/3* fusions was 5.3% in our cohort, dMMR/MSI mCRCs deem more likely to carry these fusions, making them a selected population for screening. A recent study reported a similar frequency of *NTRK* fusions in all stages of dMMR/MSI CRCs (5%).^13^ In our specific and large cohort of dMMR/MSI mCRC, we found that the dMMR/MSI *MLH1*ph and *RAS* wt CRCs represent a subpopulation in which *NTRK* gene fusions, and mostly *NTRK1* fusions, are overrepresented, reaching 47.5% (8 out of 17 patients, Table 1, Table 2 and Table 1, Table 2). These results are consistent with those from other studies including mainly non-metastatic dMMR/MSI CRCs.^13^^,^^17^^,^^19^^,^^21^^,^^23^ We reported two patients with primary resistance to ICI and peritoneal carcinomatosis who achieved complete response at 34 and 21 months of therapy with larotrectinib in the NAVIGATE trial, respectively. These cases indicate the potential of TRK inhibitors in this setting. Interestingly, these patients’ tumors harbored *TMP3-NTRK1* gene fusions, expressed loss of MLH1 expression with *MLH1* hp, and were *RAS/BRAF*^600E^ wt. Thus, our results support a two-step algorithm for molecular screening in mCRC patients, wherein routine MSI and *RAS* testing are conducted first, followed by screening with a dedicated algorithm for gene fusions only in MSI/*RAS* wt cases, particularly after ICI failure, as recently proposed by Delaye et al.^20^ However, in this latter study, the metastatic or non-metastatic status of dMMR/MSI CRC patients was non-accurate. A comprehensive testing algorithm is required to improve the feasibility and cost-efficiency from both therapeutic and medical-economics perspectives. The main techniques employed for *NTRK* fusions detection include IHC, FISH, RT–PCR, or next-generation sequencing-based, depending on the probability of *NTRK* fusion.^26^ In our series, all pan-TRK-positive cases were confirmed to harbor an *NTRK* fusion, suggesting high specificity of pan-TRK IHC in CRC.^11^ However, the sensitivity was low, as half of the FISH-positive cases were IHC-negative. Fixation conditions greatly influence TRK IHC sensitivity, and false negatives are frequent in case of non-optimal fixation.^27^ Some of the tested samples were from 1998, so the sample age could have also been a reason for the absence of the IHC signal. Moreover, interpretation of pan-TRK IHC can be challenging due to various staining patterns linked to different underlying *NTRK* gene fusions. Lower sensitivity is notably observed for TRK-C and TRK-A.^15^^,^^28^^,^^29^ Lack of TRK staining has occasionally been reported in CRCs harboring *TPM3-NTRK1* and *ETV6-NTRK3* fusions,^15^^,^^30^ as in our series. Furthermore, the high fraction of false-negative cases by IHC and by the Archer assay confirms the sub-optimal preanalytical conditions (retarded or inadequate fixation), as observed in retrospective multicentric cohorts.^25^ Consequently, FISH appears to be a more robust tool to maximize the detection of TRK alterations in this setting.

Generally, a high degree of tumor heterogeneity results from genetic instability. Our study highlighted a potential limitation of the 10%-15% FISH threshold, as observed in three cases including two LS cases. This raised the question of false FISH positivity with such low thresholds. In glioblastomas, RNA sequencing is recommended in cases with ˂30% of *NTRK* fusion-positive nuclei by FISH.^31^ Unfortunately, in our three cases, RNA sequencing was inconclusive due to poor RNA quality. Therefore, larger datasets of FISH results from dMMR/MSI CRCs should be analyzed to better choose thresholds with clinical utility for patient selection for TRK inhibitors.

In conclusion, we showed a global *NTRK* fusion incidence of 5.3% in dMMR/MSI mCRCs and revealed a markedly higher fraction of the fusion-carrying cases in the MLH1ph/*RAS* wt subpopulation. Therefore, systematic screening for *NTRK* fusions in these mCRCs is warranted. Although targeted RNAseq is the first-choice method, FISH might be more convenient in the real-life setting. If both methods are not available or fail, tumors should be tested by IHC, which needs improvement in terms of sensitivity and validation on additional similar cohorts. Specific TRK inhibitors represent effective therapeutic options for patients with *NTRK* fusion-harboring tumors after progression under ICIs.
